# The nursing staff's opinion of falls among older persons with dementia. a cross-sectional study

**DOI:** 10.1186/1472-6955-10-13

**Published:** 2011-06-20

**Authors:** Solveig Struksnes, Margareta Bachrach-Lindström, Marie Louise Hall-Lord, Randi Slaasletten, Inger Johansson

**Affiliations:** 1 Associate professor, Center of Care Research, Department of Health, Care and Nursing, Gjövik University College, 2815 Gjövik, Norway; 2 Associate Professor, Faculty of Health Sciences, Department of Medicine and Care, Division of Nursing Science, Linköping University, 58183 Linköping, Sweden; 3 Professor in Nursing, Center of Care Research, Department of Health, Care and Nursing, Gjövik University College, 2815 Gjövik, Norway; 4 Faculty of Social and Life Sciences, Karlstad University, 65188 Karlstad, Sweden

**Keywords:** Dementia, falls, nursing home, nursing care, older persons

## Abstract

The aim of this study was to describe the nursing staff's opinion of caring for older persons with dementia with the focus on causes of falls, fall-preventing interventions, routines of documentation and report and the nursing staff's experiences and reactions when fall incidents occur. A further aim was to compare these areas between registered nurses (RNs) and enrolled nurses (ENs) and staff with ≤5 and >5 years of employment in the care units in question.

**Background:**

Falls are common among older people and persons with dementia constitute an additional risk group.

**Methods:**

The study had a cross-sectional design and included nursing staff (n = 63, response rate 66%) working in four special care units for older persons with dementia. Data collection was conducted with a questionnaire consisting of 64 questions.

**Results:**

The respondents reported that the individuals' mental and physical impairment constitute the most frequent causes of falls. The findings also revealed a lack of, or uncertainty about, routines of documentation and reporting fall-risk and fall-preventing interventions. Respondents who had been employed in the care units more than five years reported to a higher degree that colours and material on floors caused falls. RNs considered the residents' autonomy and freedom of movement as a cause of falls to a significantly higher degree than ENs. RNs also reported a significantly longer time than ENs before fall incidents were discovered, and they used conversation and closeness as fall-preventing interventions to a significantly higher degree than ENs.

**Conclusions:**

Individual factors were the most common causes to falls according to the nursing staff. RNs used closeness and dialog as interventions to a significantly higher degree to prevent falls than ENs. Caring of for older people with dementia consisted of a comprehensive on-going assessment by the nursing staff to balance the residents' autonomy-versus-control to minimise fall-risk. This ethical dilemma should initiate development of feasible routines of systematic risk-assessment, report and documentation.

## Background

Fall may be defined as an unexpected event in which the person comes to rest on the ground, floor, or lower level [1]. Fall incidents and risk of falling are common problems among older groups of the population. Usually, falls do not result in medical treatment of injuries, but about 10% require hospitalisation because of fractures after falls [2,3]. Falls constitute the most important cause of death after accidents among persons over 65 years [4,5], and it is reported that the mortality rate 6 and 12 months after injury has remained essentially unchanged over the last four decades [6].

The risk of falling most often relates to the individual's health condition, such as heart- and coronary diseases, osteoporosis and gait-and balance problems. The fear of falling itself seems to result in impaired activity and subsequently increase the risk of falling [7-9]. Intervention programs aimed at intrinsic risk-factors have focused on gait training, use of hip protection and follow-up of the person's physical condition [10-14]. Environmental risk-factors, such as different obstacles and defect materials and remedies, also directly or indirectly cause falls [15,16], and a significant correlation has been found between falls and the use of certain medication [17-20]. Interventions aimed at external risk-factors are for instance environmental adjustments related to furniture, illumination and floors, change of medication and education programs for the nursing staff [10-13].

Dementia disorder constitutes an additional fall-risk [21,22]. The diagnosis relates to cognitive impairment or deficits in two or more areas of cognition, with progressive worsening of memory and other cognitive functions that have an impact on the performance of activities of daily life [23]. Considerable variation of incidence rates of falls between different types of geriatric facilities are reported [14]. With regards to people with dementia, studies report incidence rates between 4.1 and 6.2 falls per person year [15,21]. Individually targeted multi-factorial fall-risk assessment and intervention programs seem to be the most efficient intervention to prevent falls [3,12,14,24,25], but Gates et al. [26] found the evidence of this to be limited as regards primary care, community, or emergency care settings.

Nursing staff seem to have sufficient knowledge about pathological conditions which constitute fall-risk [27]. Yet preventing falls in people with impaired cognition seems to fail [17,28]. The special challenges to the nursing staff regarding the safety of residents with dementia are described [27,29], and it is suggested that this group of residents should focus on other risk factors [30]. A summary of the available research has focused on identifying the causes of falls, the effect of fall-preventing interventions, routines for risk assessment and the documentation of falls and fall injuries. There are few studies focusing on the nursing staff's experiences with falls and fall prevention [27], but it is reported that nurses feel stress related to being responsible for the residents' safety and that they strive to assess the fall-risk up against the residents' freedom of movement [29].

### Aim

The aim of this study was to describe the nursing staff's opinion of caring for older persons with dementia with the focus on causes of falls, fall-preventing interventions, routines of documentation and report and the nursing staff's experiences and reactions when fall incidents occur. A further aim was to compare these areas between registered nurses (RNs) and enrolled nurses (ENs) and staff with ≤5 and >5 years of employment in the care units in question.

## Methods

The study had a cross-sectional design. Nursing staff working in special care units for older persons with dementia were included. Permission to conduct the study was given by the section leader of all the nursing homes in the local community, and the head nurses in seven nursing homes were contacted. Four out of these agreed to participate. The inclusion criterion for the respondents was to be caregiver with permanent condition of employment. The head nurses of each care unit selected the respondents who fulfilled the inclusion criterion (n = 104).

### The questionnaire

The questionnaire developed for the study was based on previous research [14,29], in which four main areas stand out as relevant aspects on the subject of fall prevention: 1) causes of falls, 2) the nursing staff's fall-preventing interventions 3) the nursing staff's routines of documentation and report, and 4) the nursing staff's experiences and reactions related to fall incidents.

The questionnaire consisted of 64 questions. Seven background questions were related to age, education, years of experience with health care, years employed in the actual care unit, profession, percent of full-time employment and shift schedule. The following 57 questions were organised according to the four areas mentioned above.

The majority of the questions had a four-step Likert's scale with response alternatives 1) seldom, 2) sometimes, 3) often and 4) very often.

The area about *causes of falls *comprised 28 questions. Fifteen questions were about causes related to the individual. Eight questions were related to environmental factors and five questions were about the staff's organisation, priorities and assessments.

The second area was associated with *the nursing staff's fall-preventing interventions*, and included seven questions.

The third area was *routines of documentation and report*, which comprised 12 questions, out of which five were related to checking routines of remedies. The response alternatives were "yes", "no" and "do not know" in addition to one open-ended question about "how often". Three questions about routines of documentation and report had response alternatives "yes", "no" and one open-ended question about how this documentation was carried out. Three questions were related to routines of informing relatives.

The last area comprised 10 questions about *the nursing staff's experiences and reactions associated with fall incidents*. The previously described Likert's scale was used as response alternatives. One out of these questions was about the staff's conception of their own competence in fall-prevention, and had the response alternatives "yes" and "no".

For the sake of clarity, a pilot test of the questionnaire was carried out with four respondents from nursing homes other than those in question. As a result of comments from the pilot tests, minor adjustments of the questionnaire's layout were made.

### Data collection

The questionnaires were distributed and collected by the head nurses, who kept track of the response rate through a list of the staff and referred them to the researchers. After two weeks the response rate was low, n = 54 (52%). Researcher 'SS' visited the nursing homes for two reminders, and also called the head nurse to ask her if she could make a general second reminder to all the staff. These reminders resulted in nine additional respondents and a total sample of 63 (66%).

### Data analysis

Data analysis was computed with Statistical Package for the Social Sciences (SPSS) version 15 [31]. Data regarding demographics and routines of documentation and report are presented as frequencies, percentages, mean and SD. The tested areas were: what cause falls, preventive interventions, and staff reactions related to fall incidents. Differences in proportion between ENs and RNs were tested for statistical significance by Chi-square test. Mann-Whitney U-test was used to test differences between the sub-groups related to the variables of the four areas in the questionnaire.

A p-value < .05 (two tailed) was considered statistically significant.

### Ethical issues

Data were handled according to ethical guidelines for research [32,33]. The respondents were informed both orally and in writing by one of the researchers ('SS'). Voluntary participation and the right to withdraw from participation at any time was assured. Confidentiality was maintained by coding the questionnaires. The coded list was kept by the head nurse. The study was approved by The Data Inspectorate in Norway.

## Results

The results are presented according to the respondents' answers in relation to the four main areas in the questionnaire. The respondents' mean age was 44.6 years, SD 11.4, range 22-65. There were no significant differences between RNs and ENs regarding age. The respondents' remaining demographic data are presented in Table 1.

**Table 1:** Demographic data (n = 63)

	n	%
LEVEL OF EDUCATION		
Elementary	17	(27)
High school	29	(46)
College/university	17	(27)
YEARS EMPLOYED IN HEALTH CARE		
≤5	11	(17)
>5	52	(83)
YEARS EMPLOYED IN THE CARE UNIT		
≤5	34	(54)
>5	29	(46)
EDUCATION		
Unskilled nurse aid	3	(5)
Enrolled nurse (EN)	40	(63)
Registered nurse (RN)	14	(22)
Other professions	6	(10)
PERCENT OF FULL TIME EMPLOYMENT		
0-25%	6	(10)
26-50%	3	(4)
51-75%	32	(51)
76-100%	22	(35)
SHIFT SCHEDULE		
Only daytime	1	(2)
Only nights	3	(4)
Day and night	48	(76)
Day, afternoon and night	10	(16)
Only weekends	1	(2)

The majority of the respondents were ENs. Only three (5%) were unskilled nurse aids. Over 80% had worked more than five years within health care and out of these 63% (n = 25) of the ENs had more than 15 years' experience (not shown in table). Nearly 50% of the respondents had worked in the care unit in question more than five years, the RNs for a shorter period of time than the ENs (not significant). The majority of the respondents were employed for more than 50% of a full-time job, and worked both day and night shifts.

### Opinions on of what cause falls

The staff's conception of what factors precipitate falls among persons with dementia are presented in Table 2.

**Table 2:** Nursing staff's opinion of what causes falls

								Worked in the care unit in question			
CAUSING FALLS	Total n = 63		ENs n = 40		RNs N = 14		p-value^1)^	≤5 years n = 34		>5 years n = 29	
**FACTORS RELATED TO THE INDIVIDUAL**	**Mean**	**SD**	**Mean**	**SD**	**Mean**	**SD**		**Mean**	**SD**	**Mean**	**SD**

**Forgetfulness related to**											
Time, place and situation	2.2	1.1	2.3	1.1	2.1	1.0		2.2	1.1	2.3	1.1
Impaired physical condition	3.0	.8	3.0	.7	3.4	.6		3.1	.8	3.0	.7
Mental condition	2.3	.9	2.3	.9	2.1	.9		2.2	1.0	2.4	.8
How to use remedies	1.9	.9	2.1	1.0	1.6	.8		1.9	.9	2.0	1.0
That one need help with ADL	2.3	.9	2.3	.9	2.2	.9		2.2	.8	2.2	1.0
											
**Anxiety/confusion**	2.8	.9	2.9	.9	2.8	1.0		2.6	.9	3.0	.8
											
**Ability to understand and express one self**											
Ability to call for help	2.8	.8	2.8	.9	2.7	.8		2.8	.7	2.8	1.0
Ability to understand staff's instructions	2.3	.9	2.5	.9	2.2	1.0		2.2	.9	2.4	1.0
											
**Physical factors**											
Mobility	2.7	.9	2.7	.9	3.0	.7		2.7	.9	2.8	.8
Bodily built	1.9	.9	2.0	1.0	1.9	.8		1.9	.8	1.9	1.0
Body control and movements	2.7	.8	2.7	.8	2.9	.8		2.5	.8	2.9	.8
Impaired assessment of distance	2.5	.8	2.6	.9	2.5	.8		2.4	.8	2.6	.8
Dizziness	2.5	.84	2.5	.8	2.8	.9		2.6	.8	2.4	.9
Sight	2.4	.70	2.4	.7	2.4	.8		2.3	.7	2.4	.7
Hearing	2.0	.9	2.0	.9	1.8	1.1		2.0	1.0	1.9	.9
											
ENVIRONMENTAL FACTORS											
**Physical environment**											
Unsurveyable locals and long distances	1.6	.7	1.6	.8	1.5	.7		1.6	.7	1.6	.8
Colours and materials on floors	1.4	.7	1.5	.8	1.3	.5		1.3	.6	1.6	.8
Carpets, furniture and equipment	1.3	.6	1.4	.6	1.1	.3	.051	1.4	.6	1.3	.5
Illumination	1.3	.5	1.2	.4	1.4	.5		1.3	.6	1.3	.4
Wet floors	1.3	.6	1.2	.5	1.4	.7		1.4	.7	1.1	.4
											
**Psycho-social environment**											
Noise and disturbances	1.5	.7	1.5	.7	1.5	.7		1.5	.7	1.5	.8
Disturbing co-residents	2.0	.8	2.0	.8	2.2	.8		1.9	.8	2.1	.8
Aggressive and annoying co-residents	2.0	.8	2.0	.8	2.2	.9		1.9	.8	2.1	.7
											
**Staff organisation and priorities**											
Number of staff	2.4	.9	2.4	.9	2.7	.8		2.2	.9	2.6	.9
Planning and prioritizing work	1.5	.7	1.6	.6	1.6	1.0		1.5	.7	1.5	.7
Insufficient knowledge of the residents risk behavior	1.4	.6	1.4	.5	1.7	.9		1.4	.7	1.5	.5
Discovering fall risk too late	1.6	.6	1.7	.7	1.5	.5		1.6	.6	1.7	.7
Prioritizing the residents' integrity and autonomi	1.9	.8	1.8	.7	2.4	.9	.023	1.9	.9	1.9	.7

^1) ^Significance test Mann Whitney U-test

The nursing staff reported the highest mean-scores related to the individuals' mental and physical impairment. The staff-resident ratio was sometimes associated with falls, but the staffs' knowledge, planning and priorities of the work were seldom reported to cause falls.

RNs emphasised the residents' freedom of movement as a risk-factor to a significant higher degree than ENs. Respondents with longer experience in the care units reported to a significantly higher degree than less experienced staff that colours and material on floors could cause falls. This sub-group also tended to emphasise number of staff as a cause of falls to a higher degree than less experienced staff (not significant).

### Fall-preventing interventions

The respondents chose different kinds of interventions to reduce and prevent fall-risk and fall incidents, as shown in Table 3.

**Table 3:** Nursing staff's choice of fall-preventing interventions

								Employed in the care unit in question			
INTERVENTIONS	Total n = 63		ENs n = 40		RNs n = 14		p-value ^1)^	≤5 yeras n = 34		>5 years n = 29	
	**Mean**	**SD**	**Mean**	**SD**	**Mean**	**SD**		**Mean**	**SD**	**Mean**	**SD**

Conversation and closeness	3.4	.6	3.3	.5	3.7	.5	.015	3.4	.6	3.4	.5
Music	2.8	.8	2.7	.8	2.9	.9		2.6	.8	3.0	.8
Extra supper meal	2.8	.8	2.8	.8	2.9	.9		2.7	.7	3.0	.8
Diverting activities	3.0	.7	2.9	.7	3.1	.8		3.0	.8	2.9	.6
Assistance with personal hygiene	3.4	.8	3.5	.7	3.8	.5		3.3	.9	3.6	.6
Administrating sedatives (RN)	2.0	.8	-	-	2.2	.7	-	1.9	.8	2.3	.8
Ask for ordination of sedatives (EN)	1.7	.8	1.6	.7	-	-	-	1.8	.9	1.7	.7

**Scale: **1 = Seldom, 2 = Sometimes, 3 = Often, 4 = Very often.. ^1) ^Significance test Mann Whitney U-test

The most frequent interventions were conversation and closeness and assistance with personal hygiene. Significant differences were noted between RNs and ENs regarding some fall-preventing interventions. RNs chose conversation and closeness to a significantly larger extent than the ENs. Years of experience in the care unit in question made no significant difference regarding the choice of interventions. However, a nursing staff with a longer experience in health care in general (>5 years) used assistance with personal hygiene as a fall prevention significantly more often than colleagues with less experience (Z = 2.242, p = .03. Not shown in table).

The respondents reported that 44% chose freedom of movement often, 17% very often, 19% seldom and 4% very seldom. Control was preferred often by 48% very often by 12%.

### Routines of documentation and report

The majority (95%) of the respondents reported that all fall incidents were registered, and that fall incidents were discussed in the care unit in connection with the oral reports. Registrations were done in the individual care plans, but also in special registration forms. The respondents had different views about documentation of fall-risk and fall-preventing interventions. Approximately 60% answered that the care unit documented these data on a regular basis. There was no significant difference between the compared sub-groups regarding these questions.

Information to relatives about falls with no visible injuries were given often (30%) or very often (21%) according to the respondents. Visible injuries increased the frequency of information to relatives, as 33% reported "often" and 64% "very often". Severe injuries were very often (95%) reported to relatives, and there was no significant difference in opinion between the sub-groups on this matter.

Out of the 63 respondents, between 32-51% did not know if different types of remedies were controlled.

### Nursing staff's experiences and reactions to fall incidents

The respondents report with concern to this area is shown in Table 4.

**Table 4:** Nursing staff's experiences and reactions

								Worked in the care unit in question			
											
	Total n = 63		ENs n = 40		RNs n = 14		p-value^1)^	≤5 years n = 34		>5 years n = 29	
	**Mean**	**SD**	**Mean**	**SD**	**Mean**	**SD**		**Mean**	**SD**	**Mean**	**SD**

Being present at fall situation last three months	1.3	.6	1.3	.5	1.3	.6		1.3	.5	1.4	.6
Prevented fall last three months.	1.6	.6	1.6	.6	1.6	.7		1.6	.7	1.6	.6
Experienced to not hinder fall last three months	1.2	.4	1.2	.5	1.2	.4		1.2	.5	1.1	.3
Takes long time before falls are discovered	1.2	.4	1.1	.3	1.2	.4	.013	1.3	.5	1.2	.4
Experienced stress in relation to fall incidents	1.2	.5	1.2	.4	1.3	.5	.050	1.2	.4	1.3	.5
Experienced unease in relation to fall incidents	1.4	.6	1.4	.6	1.4	.6		1.3	.5	1.5	.7
Experienced guilt in relation to fall incidents	1.2	.4	1.2	.5	1.2	.4		1.2	.4	1.2	.5
To ensure the persons freedom of movement is important to prevent falls	2.7	.8	2.6	.8	2.7	.8		2.8	.9	2.7	.8
Interventions to control the residents are important to prevent falls	2.6	.9	2.5	.8	2.6	.8		2.5	.9	2.7	.8

**Scale: **1 = Seldom, 2 = Sometimes, 3 = Often, 4 = Very often.. ^1) ^Significance test Mann Whitney U-test

The respondents in total reported that it "seldom" took a long time before fall incidents were discovered, but RNs to a significantly higher degree experienced that it took longer time.

Nearly all respondents (90%) reported that they had sufficient competence in fall prevention, and seldom felt stress, unease or guilt related to being present in fall situations. However, RNs tended to feel stress in relation to fall situations to a higher degree than ENs (not significant).

## Discussion

The study aimed at describing the nursing staffs' opinion of what causes falls, documentation and report related to fall-risk and fall incidents, interventions and their experiences and reactions associated with working with older people with dementia. The discussion of the results is presented according to these four areas.

*Causes of falls *are, according to the respondents, most often related to the individual's condition. Four causes stand out as the most frequent: forgetfulness related to physical impairment, impaired mobility, anxiety, and inability to call for help. These results are in line with other studies [14,15,34,35]. Impaired short-time memory makes people with dementia forget their physical impairment, and falls can occur when they are getting up from the bed or a chair [17,29,36].

Those who can get up from a chair, but cannot stand upright or sit down unaided are particularly exposed to fall-risk [16,17].

Anxiety was reported to "often" cause falls among the respondents. This results are in line with other studies showing that anxiousness and confusion are symptoms that evidently precede falls [22,34].

Hyperactive, restless and paranoid behaviour may characterise some persons with dementia, and it is reported that residents who have the highest risk of falls are wanderers [22]. As opposed to this the nursing staff in this study did not explicitly report wanderers as a risk group.

Environmental factors were not reported as a frequent cause of falls by the respondents. For comparison, studies suggest that external, physical factors are estimated to precipitate about 8% of falls in residential care facilities [17], while others have found that 14,5% of falls are associated with environmental factors [16]. A Swedish study indicates that well-planned furnishing and the use of colour to achieve a plain, clearly-defined environment reduce fall incidence in residential care facilities [37]. An explanation to the fact that the respondents do not emphasise this issue may be that the facilities in the nursing homes in question are modern, surveyable and well customised for persons with dementia.

Disturbing behaviour of co-residents "sometimes" precipated falls according to the nursing staff in question. Previous studies do not point out mistreatment or disturbances from other residents as a significant precipitant to falls [17], but if these kinds of environmental factors are associated with anxiety and anger they could increase the odds of inducing falls, according to French et al. [22].

The respondents seem to emphasise staff-to-resident ratio as an indirect cause of falls to a higher degree than how the staff plan and organise their work. These factors probably interact. According to a study by Pellfolk et al. [36] there is no difference between fallers and non-fallers regarding staff-to-patient ratio. However, the factors communication, management policies and teamwork are considered to be of importance for successful fall management [38]. Lack of staff influences the staff's priorities in daily work and may force them to organise in ways that are efficient regarding the basic tasks in the care unit. To find time to discuss systematic routines of fall prevention and implement these in the care units may be a challenge in a busy working day.

The majority of the respondents in this study reported having sufficient knowledge about fall prevention. However, there were differences between the compared groups regarding their reports on what causes falls. As previously described, respondents with longer experience are significantly more aware of the condition of the floors as directly or indirectly causes of falls. According to Lundin-Olsson et al. [39] nursing staff's assessment of fall-risk seems to be fairly accurate in predicting falls in residential care facilities. This may indicate that the attention to environmental risk-factor evolves with years of experience, rather than educational level.

The two most frequent *fall-preventing interventions *reported by the respondents were conversation and closeness and assistance with personal hygiene. Impaired cognition and ADL function are evidently risk-factors [34]. It also seems that the majority of falls occur in the residents' own rooms, unwitnessed [36]. Thus, the nursing staff's choice of interventions seem appropriate.

Comparisons between the sub-groups showed significant differences regarding what they preferred as adequate interventions. In general, staff with more experience in health services chose assistance with personal hygiene as an intervention to a significantly higher degree than less experienced staff. RNs chose conversation and closeness as an intervention to a significantly higher degree than the ENs. The results do not describe whether the choice of interventions were based on evidence or the staff's personal experiences, but in this case education seems to make a difference in how fall prevention is carried out.

Although nearly all respondents reported that they had sufficient competence in fall prevention, the findings did not describe a systematic strategy with multi-factorial and individually-targeted risk assessment and intervention, which is recommended to reduce fall rate in an institution [1,3,14,40]. However, the multiple experience-based and single-targeted interventions conducted by the nursing staff may be important knowledge, as a basis for the development of more feasible routines to prevent falls.

How to ensure a qualified and co-ordinated nursing staff may be an interesting subject of research. So far there is no strong evidence that educational programs for nursing staff have any significant impact on reducing fall rate among people with dementia [3,14]. Implementation of models of interventions have proved to be difficult, and it is suggested that the most successful strategy appears to be changing attitudes of nurses in order to increase the focus on fall prevention [41].

*Routines of documentation and report *was an area where the respondents had considerable variation in perceptions. The disagreement on this issue may be understood as lack of routines or inadequate following-up of existing routines. The impact of multi-factorial risk-assessment and interventions seems to presuppose a comprehensive co-ordinated group of health workers [14]. The respondents in this study discussed fall incidents on a regular basis, but only 60% register fall-risk in the individual care plans. As pointed out in different studies the first step in the prevention of falls in nursing care is systematic, individual risk assessment [14,42]. This presupposes adequate communication between the nursing staff within a care unit, and cooperation between different professions and units in the nursing home. To obtain this, a sufficient number of qualified staff who are organised with feasible routines of documentation and reports are required. Lack of routines of documentation and report related to fall-risk assessment may cause an inattentive nursing staff. A consequence of this may be that fall-preventing interventions are not planned or conducted before it is too late.

*Nursing staffs' experiences and reactions when attending fall situations *is a subject that has been given little attention in other studies. In the present study ENs and RNs only seldom experienced being present at fall situations or preventing falls. When falls occurred, the majority of the respondents seldom felt stress, unease or guilt related to attending fall situations, but RNs feel stress to a significantly higher degree than ENs. These results oppose some studies which describe nursing staffs' experience of stress related to being responsible for the residents' safety [27,29]. However, when one third of the respondents "sometimes" or "often" felt unease when they experienced these situations, the findings should be of interest for further exploration. Rush et al. [43] describes acute nurses' experience with resident falls, and found that this created considerable stress for nurses which prompted them to use a range of coping strategies. Knowing that the residents are safe has the potential to resolve the tension between patient safety and independence [43]

Although the nursing staff in this study considered that prioritising the residents' freedom of movement "sometimes" or "often" cause falls, they prioritised autonomy and freedom as often as they chose a strategy of control. One type of protection and control is physical restraint. It is reported that nursing staff often use restraints to protect these residents, although the effectiveness of these interventions has not been proved [44]. Previous studies have found that there is a comprehensive on-going assessment by the nursing staff to balance the residents' autonomy-versus-control in order to minimise fall-risk [29]. It is argued that in this context there are some essential values and norms that should be observed in an ethical evaluation of physical restraint [13,45].

The RNs to a significantly higher degree prioritised the residents integrity and autonomy as a risk factor. These differences may be explained by the fact that years of experience is important for developing skills in clinical observation and assessment, while nurse education aims at developing a higher degree of ethical reflection, evidence-based knowledge and critical thinking.

### Methodological issues

The four nursing homes were quite similar with regards to staff-resident ratio, buildings and other enviromental factors. In total the residents in these care units for persons with dementia represented a wide variation related to physical and cognitive impairment. Thus the findings represent a representative cross-sectional picture of the nursing staffs' challenges related to caring for people with dementia at risk of falling.

The response rate was 66%, which is acceptable for a survey [46]. Study limitations are related to a small sample and the sample distribution (ENs: n = 40, RNs: n = 14). Further studies in larger scale are required.

The questionnaire was developed according to previous research on the subject [29]. Hence the use of sedatives is not listed as a possible cause of falls, but is related to the nursing staffs' interventions. On the other hand, side effects of sedatives, such as dizziness and gait or balance problems, are listed as options in the questionnaire.

The questions about checking remedies had a low response rate (27%) and were insufficiently filled in by some of the respondents. Whether this is related to the formulation of the questions or a general doubt regarding these routines is difficult to know. However, the findings indicate divergent opinions within the nursing staff that may call for action with regards to routines in the care units in question.

To validate the questionnaire the research team and head nurses participated in the formulation, and pilot tests were conducted. Still, further testing of the instrument is required for validity and reliability.

## Conclusions

Individual factors were the most common causes of falls according to the nursing staff. The nursing staff used a wide spectre of single-targeted interventions to prevent falls and RNs used relation-based interventions to a significantly higher degree than ENs. The respondents report a comprehensive on-going assessment by the nursing staff to balance the residents' autonomy-versus-control in order to minimise fall-risk.

The findings should initiate the development of feasible routines of systematic risk-assessment, report and documentation. Further, regular staff meetings where basic values and strategies in the care of demented persons are discussed should be established as a routine.

## Competing interests

The authors declare that they have no competing interests.

## Authors' contributions

SS participated in the design of the study and development of the questionnaire. SS was responsible for data collection and statistical analysis. SS drafted the manuscript and was responsible for editing the final manuscript. MBL participated in the design of the study, development of the questionnaire, and reading, commenting and final approval of the manuscript. MLHL participated in the design of the study, development of the questionnaire, supervision of statistical analysis and reading, commenting and final approval of the manuscript. RS participated in the design of the study, development of the questionnaire, and reading, commenting and final approval of the manuscript. IJ participated in the design of the study, development of the questionnaire and supervision of statistical analysis. IJ supervised the drafting of the manuscript, and read, commented and gave the final approval of the manuscript.

## Pre-publication history

The pre-publication history for this paper can be accessed here:

http://www.biomedcentral.com/1472-6955/10/13/prepub
